# Using analogy to learn about phenomena at scales outside human perception

**DOI:** 10.1186/s41235-017-0054-7

**Published:** 2017-03-20

**Authors:** Ilyse Resnick, Alexandra Davatzes, Nora S. Newcombe, Thomas F. Shipley

**Affiliations:** 1 Department of Psychology, Penn State University Lehigh Valley, 2809 Saucon Valley Road, Center Valley, PA 18034 USA; 2 Department of Earth and Environmental Sciences, Temple University, Philadelphia, PA 19122 USA; 3 Department of Psychology, Temple University, Philadelphia, PA 19122 USA

**Keywords:** Analogy, Magnitude, Progressive alignment, Corrective feedback, STEM education

## Abstract

Understanding and reasoning about phenomena at scales outside human perception (for example, geologic time) is critical across science, technology, engineering, and mathematics. Thus, devising strong methods to support acquisition of reasoning at such scales is an important goal in science, technology, engineering, and mathematics education. In two experiments, we examine the use of analogical principles in learning about geologic time. Across both experiments we find that using a spatial analogy (for example, a time line) to make multiple alignments, and keeping all unrelated components of the analogy held constant (for example, keep the time line the same length), leads to better understanding of the magnitude of geologic time. Effective approaches also include hierarchically and progressively aligning scale information (Experiment 1) and active prediction in making alignments paired with immediate feedback (Experiments 1 and 2).

## Significance statement

Many fundamental science, technology, engineering, and mathematic (STEM) phenomena occur at extreme scales that cannot be directly perceived. For example, the Geologic Time Scale, discovery of the atom, size of the universe, and rapidly developing field of nanotechnology are all based on phenomena occurring at scales that humans cannot directly experience. Unfortunately, novices have trouble reasoning about phenomena outside human perception, at least in part, because they do not understand the relative magnitudes at these scales. Across two experiments we develop and test two successful instructional analogies designed to teach geologic time. These findings add to our theoretical understanding of how people reason about scale, and the role of analogical reasoning and active prediction in learning. We find that reasoning about different kinds of magnitude (that is, temporal and abstract) at different scales (that is, inside and outside human perception) have shared properties, and that people are able to use a spatial representation of temporal magnitude to develop a more accurate understanding of geologic time. Our findings suggest that structural alignment and having multiple opportunities to make alignments are critical in supporting reasoning about scale, and that the progressive and hierarchical organization of scale information may provide salient landmarks for estimation. Finally, we found that active prediction and corrective feedback are valuable in fostering a linear representation of magnitude. These findings have practical implications, as they can guide development of supports for the acquisition of reasoning at extreme scales, which is an important goal in STEM education.

## Background

### How can we improve students’ reasoning about large magnitudes?

Skills in reasoning about size and scale are central to performance across STEM disciplines (for example, Hawkins, 1978; Tretter, Jones, Andre, Negishi, & Minogue, 2006). Many fundamental science, technology, engineering, and mathematic (STEM) phenomena occur at extreme scales that cannot be directly perceived. For example, a core geologic concept is that geologic events can last billions of years (for example, the Earth formed approximately 4.6 billion years ago). Reasoning about geologic time allows geologists to reconstruct the surface conditions of ancient Earth, produce an accurate time line of Earth’s history, and understand the imperceptibly slow processes that have led to the current environment. Practically, understanding geologic time allows people to reason about the sustainability of non-renewable resources and the consequences of anthropogenic climate change. Given the importance of reasoning about scale, it should be no surprise that the National Research Council in *A Framework for K-12 Science Education* (National Research Council, 2011) and the American Association for the Advancement of Science in *Benchmarks for Science Literacy* (American Association for the Advancement of Science, 1993) identified “size and scale” as fundamental scientific concepts, and suggested them as a unifying theme in science education.

Unfortunately, novices have trouble reasoning about phenomena outside human perception. Although novices are sometimes reasonably accurate at ranking phenomena in a correct sequence, they have difficultly comparing the magnitude between phenomena at extreme scales (for example, Delgado, Stevens, Shin, Yunker, & Krajcik, 2007; Jones, Tretter, Taylor, & Oppewal, 2008; Libarkin, Anderson, Dahl, Beilfuss, & Boone, 2005). For example, although students are fairly accurate in identifying a correct sequence of events in Earth’s geologic history (Trend, 2001), they fail to understand the magnitude of time between events (Tretter *et al*., 2006).

Analogy is potentially valuable for learning and reasoning about phenomena outside human perception because such phenomena cannot be directly experienced (Jones, Taylor, & Broadwell, 2009). Analogy refers to a process of aligning structural similarities between a base concept and a target concept (Gentner, 1983). In fact, analogy is frequently used to teach phenomena at extreme scales (for example, Clary & Wandersee, 2009; Petcovic & Ruhf, 2008; Semken *et al*., 2009; Sibley, 2009). Unfortunately, even with a wide range of analogies, students struggle to comprehend phenomena outside human perception (for example, Delgado *et al*., 2007; Jones *et al*., 2008; Libarkin *et al*., 2005). Moreover, analogies can mislead students (Brown & Salter, 2010; Duit, 1991). For example, geologic time is often represented using a spatial analogy that compresses the time before life on Earth becomes relatively more complex. Although functional, learning from this nonlinear representation can mislead students into thinking that biologic events occurred very early in the Earth’s history (Resnick, Davatzes, Newcombe, & Shipley, 2016; Resnick, Newcombe, & Shipley, 2016).

Different kinds of spatial analogies may elicit different kinds of cognitive barriers to aligning extreme scales with human scales (for review see Resnick, Davatzes, *et al*., 2016). For example, a common analogy is to map an extreme scale (for example, Earth’s history) onto a spatial structure, such as the Eiffel Tower (Clary & Wandersee, 2009). However, without knowledge of the base concept (How tall is the Eiffel Tower?), it is difficult to identify corresponding relations between the base concept and target concept (for example, Gentner, 1983; Kotovsky & Gentner, 1996). It can also be difficult to identify the relevant relations to align if the base concept and target concept are different in many ways (Gentner, 1983; Gentner, 2001; Markman & Gentner, 1996, 1997; Kokinov & French, 2003). For example, Earth’s history is also commonly mapped onto a 24-hour clock. However, this analogy contains at least two differences in addition to differences in magnitude (24 hours versus billions of years): clocks are cyclical whereas Earth’s history is linear, and clocks are composed of equal temporal divisions whereas geologic time comprises unequal temporal divisions based on major geologic events. Thus, students may not be able to identify the appropriate analogy to make and, subsequently, draw incorrect conclusions (Brown & Salter, 2010; Gentner, 1983). In this instance, students may erroneously believe that, just like the 24-hour clock, periods of Earth’s history are also evenly spaced, and fail to make the appropriate analogy between relative magnitudes of time between events.

A review of analogical reasoning literature offers three techniques that may be useful in the development of effective analogies for phenomena at extreme scales. First, the base concept and target concept should be *structurally aligned*; that is, as similar as possible with just one “alignable difference” (Goldstone, 1994; Markman & Gentner, 1993a, 1993b; Medin, Goldstone, & Gentner, 1993). An alignable difference is a common relation shared by the base concept and target concept which differs along one dimension. In the case of aligning an extreme scale with a human scale, both scales should be constructed in the same format (for example, a linear number line), with the only difference being magnitude.

However, because the difference in magnitude between extreme and human scales is so vast, it may not be possible for the base concept and target concept to be structurally aligned. Thus, a second technique to align very different concepts is called *progressive alignment* (Kotovsky & Gentner, 1996; Thompson & Opfer, 2010). Using progressive alignment, an analogy may consist of more than one analogical step, beginning with a comparison of a base concept and a highly similar intermediate concept. Comparing two very similar concepts as an intermediate analogical step will help extend the analogy to the subsequent alignment of increasingly unfamiliar concepts (Gentner & Namy, 2006). For example, instead of mapping Earth’s history directly onto a spatial time line, the analogy may first map a human lifespan, and increase the amount of time the spatial time line represents in separate analogies (for example, American history, human evolution, and so on) until all of Earth’s history is included in the spatial time line. In learning about scale information, progressive alignment may alleviate conceptual dissimilarity by providing structural alignment across smaller increases of scale (Resnick, Davatzes, *et al*., 2016).

Finally, a third technique is called *hierarchical alignment* (Resnick, Newcombe, *et al*., 2016). Hierarchical alignment advocates the hierarchical organization of all analogical steps within each new analogy to highlight common relational structures between the base, intermediate, and target concepts. In the Earth’s history example, the learner would identify where each previous division in time (for example, human lifespan) would be located relative to the new spatial time line (for example, American history). This process of hierarchical alignment provides salient internal anchor points, which emphasize corresponding relations between each analogical step (Resnick, Newcombe, *et al*., 2016). Hierarchical alignment specifically supports learning about phenomena outside human perception by highlighting the proportional relation between magnitudes across multiple scales.

In addition to the principles of analogical reasoning discussed above, immediate (Coulter & Grossen, 1997) and corrective (Hao, 1991; Sharpe, Lounsbery, & Bahls, 1997) feedback has also been found effective in increasing student learning. In particular, corrective feedback has been found to be effective for learning about unfamiliar magnitudes in young children (Thompson & Opfer, 2010). Even a single trial of feedback can increase estimation accuracy by providing learners with a salient anchor (Opfer & Siegler, 2007; Opfer & Thompson, 2008; Opfer, Thompson, & Kim, 2016; Thompson & Opfer, 2008).

### The current studies

In the current studies we ask whether analogies can foster accurate reasoning about phenomena at scales outside human perception and, if so, what the most efficient and effective techniques in teaching scale information are. While the instructional analogies developed here could be designed for use in teaching any magnitude-based context, we examine analogical reasoning in the context of a large temporal magnitude: geologic time.

Over two experiments we develop two instructional analogies using a combination of structural alignment, progressive alignment, and hierarchical alignment. Of importance, all three techniques provide multiple opportunities to practice making relevant analogies. Thus, both Experiments 1 and 2 examine the efficacy of providing multiple opportunities to align geologic time to a spatial linear representation using structural alignment to improve understanding and reasoning about geologic time. Across both experiments, students are also provided with corrective feedback. A key difference between Experiments 1 and 2 is that Experiment 1 also assesses the benefit of hierarchical and progressive alignment, whereas Experiment 2 assesses structural alignment and corrective feedback alone, without progressive or hierarchical alignment, in an effort to devise a more time-efficient means of intervention.

## Experiment 1

The *hierarchical alignment activity*, an instructional spatial analogy that employs structural, progressive, and hierarchical alignment, increased accuracy reasoning about temporal and spatial magnitudes in a lab-based setting (Resnick, Newcombe, *et al*., 2016). Here, we assess the efficacy of the hierarchical alignment activity in a classroom setting. Learners begin by aligning a familiar scale to a linear spatial representation (a number line). They are then provided with multiple opportunities to align increasingly larger and unfamiliar scales to the spatial linear representation (progressive alignment). Structural alignment is maintained by keeping the length of space the same for each analogical step; only the magnitude of time changes. In this activity, every time students align a new temporal scale to space, they are also asked to locate all previous scales relative to the current scale (hierarchical alignment). After completing each intermediate analogy, learners are provided with corrective feedback.

The hierarchical alignment activity is contrasted with a conventional spatial analogy for geologic time called a stratigraphic column. A stratigraphic column is a spatial representation of the vertical location and age of rock units. Of importance, the hierarchical alignment activity and the stratigraphic column activity differ only in the use of analogical reasoning principles (structural, progressive, and hierarchical alignment) in their presentation of geologic time. Students in both activities learned the names and magnitudes of geologic time periods by aligning time to space. Thus, if the hierarchical alignment activity fosters more accurate understanding and reasoning about geologic time than the conventional stratigraphic column activity, this would suggest the importance of structural, progressive, and hierarchical alignment in fostering more accurate reasoning about phenomena at scales outside human perception.

## Methods

### Participants

The participants were 107 students (49 in the experimental group and 58 in the control group) enrolled in an undergraduate introductory-level geoscience course at a large urban university. Although the demographics of the participants could not be obtained, students were randomly assigned to either condition, which accounts for any individual differences. Demographic information was obtained in subsequent semesters of this same course during Experiment 2. While there may be some variation from semester to semester in demographic composition, Table 1, which contains information gathered in Experiment 2, provides characteristic demographic information of this general education course aimed primarily at non-majors. The geoscience course had twice weekly lectures and a weekly laboratory period. All lectures were given by the same faculty member; the students were divided into eight sections for the laboratory period. One teaching assistant (TA) covered four sections and two TAs covered two sections each.

**Table 1:** Demographics of enrollment by class and condition in Experiment 2

	Frequency (%)					
	Clicker feedback			Linear visualization		
	Fall 2011 (Control)	Spring 2012 (Intervention)	Fall 2012 (Intervention)	Fall 2011 (Control)	Spring 2012 (Intervention)	Fall 2012 (Intervention)
Age (years)	μ = 22.1, σ = 1.4	μ = 21.7, σ = 1.9	μ = 20.0, σ = 1.5	μ = 22.72, σ = 1.9	μ = 21.7, σ = 1.3	μ = 19.8, σ = 3.8
Sex						
Male	45 (43.3)	71 (59.7)	59 (49.6)	55 (45.8)	44 (41.5)	49 (44.1)
Female	59 (56.7)	48 (40.3)	60 (50.4)	65 (54.2)	62 (58.5)	62 (55.9)
Race						
White/Caucasian	81 (77.9)	89 (74.8)	84 (70.6)	82 (68.3)	67 (63.3)	80 (72.1)
African-American	10 (9.6)	8 (6.7)	10 (8.4)	13 (10.8)	12 (11.3)	12 (10.8)
Asian	4 (3.8)	8 (6.7)	5 (4.2)	7 (5.8)	6 (5.7)	6 (5.4)
Hispanic	4 (3.8)	5 (4.2)	12 (10.1)	5 (4.2)	10 (9.4)	6 (5.4)
Not identified	4 (3.8)	8 (6.7)	7 (5.9)	7 (5.8)	8 (7.5)	3 (2.7)
Other	1 (1.0)	1 (0.8)	1 (0.8)	6 (5.0)	3 (2.8)	4 (3.6)
Education level						
Freshman	16 (15.4)	21 (17.6)	26 (21.8)	29 (24.2)	9 (8.5)	49 (44.1)
Sophomore	54 (51.9)	49 (41.2)	45 (37.8)	42 (35.0)	48 (45.3)	38 (34.2)
Junior	27 (26.0)	33 (27.7)	28 (23.5)	35 (29.2)	29 (27.4)	14 (12.6)
Senior	7 (6.7)	9 (7.6)	17 (14.3)	12 (10.0)	14 (13.2)	8 (7.2)
5+	0 (0.0)	0 (0.0)	3 (2.5)	2 (1.7)	6 (5.7)	2 (1.8)
Degree						
Bachelor of Arts	70 (67.3)	62 (52.1)	81 (68.1)	56 (46.7)	58 (54.7)	51 (45.9)
Bachelor of Science Education	6 (5.8)	12 (10.1)	7 (5.9)	13 (10.8)	11 (10.4)	14 (11.7)
Bachelor of Business	5 (4.8)	23 (19.3)	20 (16.8)	21 (17.5)	13 (12.3)	30 (27.0)
Bachelor of Science	16 (15.4)	18 (15.1)	8 (6.7)	20 (16.7)	14 (13.2)	14 (12.6)
Other Bachelor degree	7 (6.8)	4 (2.5)	3 (2.5)	10 (8.3)	9 (7.5)	3 (2.7)
Non-degree	0 (0.0)	1 (0.8)	0 (0.0)	0 (0.0)	1 (0.9)	0 (0.0)

Note: Percentages do not sum to one hundred percent because not all students disclosed demographic information

### Intervention activity

**Fig. 1:**
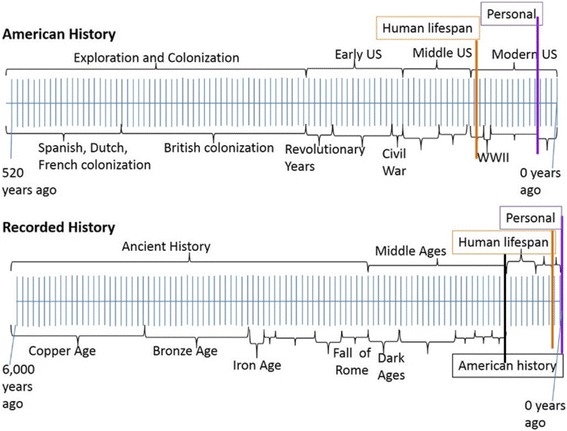
Example of two time lines from the hierarchical alignment activity. Note: These examples include two previous time lines (personal and human lifespan) not shown. *WWII* World War Two

In the hierarchical alignment activity, students aligned time to space (a 1-meter ruler) beginning with a familiar scale: their own personal time line. The students then aligned successively longer intervals of time to the same 1-meter space. Ten time lines were constructed, each ending at the present day and extending backwards in time to include: personal history, an average human lifespan (from 75 years ago), American history (520 years ago), recorded history (5512 years ago), human evolution (6 million years ago), Cenozoic Period (65 million years ago), Phanerozoic Eon (542 million years ago), Proterozoic Eon (2.5 billion years ago), Archean Eon (3.8 billion years ago), and Hadean Eon (4.6 billion years ago – the full Geologic Time Scale). To populate and relate each time line, students indicated the time line’s length, located specific events, and located where all previous time lines would begin on the current time line (see Fig. 1). In order to determine where specific events and previous time lines were located the students had to calculate how many years each centimeter represented, and then how many centimeters were needed to represent a given event or time line. The students made these calculations using two equations, which were provided. Students received corrective feedback as required to make accurate time lines.

### Control activity

In the control activity, students also aligned time to space by completing a stratigraphy laboratory. Stratigraphy is a branch of geology concerned with the order, relative position, and ages of rock layers (strata). During the stratigraphy laboratory, students learned about the age and distribution of rock types and the types of environments in which those rocks are formed by making and examining stratigraphic columns. A stratigraphic column is a spatial representation of the vertical location and age of rock units.

Of importance, stratigraphic columns involve aligning geologic temporal information to space. Thus, students in both the intervention and control conditions received practice aligning geologic time to space and exposure to magnitude information. The important difference between the intervention and control conditions is the use of structural, progressive, and hierarchical alignment in the presentation of magnitude information.

### Measures

To access students’ understanding of geological scales, students were assessed on three questions and four number line estimations that were added to their regularly scheduled laboratory examination. Two questions assessed understanding of the magnitude of geologic time. The first item, referred to here as the "Geoscience Concept Inventory question," was from the Geoscience Concept Inventory. The Geoscience Concept Inventory is a multiple choice assessment instrument of geoscience understanding, which has been validated and is unbiased relative to demographic variables (Libarkin & Anderson, 2006, 2007). The Geoscience Concept Inventory question is commonly used to assess an individual’s understanding of geologic time on a linear scale (for example, Libarkin *et al*., 2005; Libarkin & Anderson, 2007; Petcovic & Ruhf, 2008; Teed & Slattery, 2011). This item contains five multiple choice response options, shown as five time lines with the same four geologic events in different locations. Four of the time lines represented common student misconceptions (response option A – life occurred when Earth formed, option B – humans and dinosaurs coexisted, option C – dinosaurs appeared much earlier than they did, option E – all life formed at the beginning of Earth’s history), and one time line showed the events in the correct relative locations (option D). Students were asked to choose the most correct time line. Incorrect response options A and B reflect relatively small magnitude errors (that is, the magnitude of error is less than 1 billion years) while incorrect response options C and E reflect relatively large magnitude errors (that is, the magnitude of error is greater than 2 billion years).

The second item assessing understanding of geologic magnitude, referred to here as the Geologic Time Scale diagram question, is a measure of geologic time developed for use with middle school students as part of a large-scale project being conducted by the 21st Century Center for Research & Development in Cognition & Science Instruction (Barghaus & Porter, 2010). This item is a multiple choice item that requires students to identify which duration-based statement is true using a conventional diagram of the Geologic Time Scale. Two of the incorrect response options reflect incorrectly reading the direction of time (response option C – The Jurassic Period ended 205 million years ago; response option D – The Pre-Archean Eon is the most recent time span). The correct choice is option A (The Proterozoic Eon lasted much longer than the Phanerozoic Eon). While numerical information is provided in the diagram, the correct choice may not be obvious to novices in the standard diagram because the spatial intervals of the eons do not proportionally correspond to their temporal lengths. This type of compressed representation is how the Geologic Time Scale is typically depicted, and serves a functional purpose in the field. In past work the most commonly chosen incorrect response was a statement that is consistent with the visible spatial intervals (response option B – The Phanerozoic lasted much longer than the Proterozoic).

The participants also completed a knowledge-based question that did not require an understanding of relative magnitude. Thus, this item served as a control for potential group differences (for example, motivation). This knowledge-based question was taken from the 21st Century Center for Research & Development in Cognition & Science Instruction study (Barghaus & Porter, 2010), and asked participants to identify when mammals were the dominant land animal.

A fourth kind of assessment examined transfer effects to estimation of large abstract numerical magnitude. Here we use number line estimations. Siegler and Booth (2004) noted that number line estimations are thought to be ecologically valid, as students often make number lines in class. A 2-year longitudinal study by Jordan *et al*. (2013) found performance on a number line estimation task to have a high internal consistency (Cronbach’s alpha 0.89). Students were presented with four horizontal number lines. The horizontal number lines were 10 cm long, with the right labeled 0 and the left labeled 4.6 billion. Students were asked to locate the following numerical values on the respective number lines in the following order: 230 million, 65 million, 3.5 billion, and 6 million. These magnitudes were chosen to be a numerical analog to the Geoscience Concept Inventory item (humans appear, 6 million years ago; dinosaurs disappear, 65 million years ago; dinosaurs appear, 230 million years ago; and life appears, 3.5 billion years ago). These number line estimations are abstract because no units (time or space) were indicated.

### Procedure

Prior to the intervention, the researcher met with the main instructor and TAs. The TAs described their prepared lessons on geologic time, and were instructed to not change their lessons in anyway. The overarching aim of the experiment was then described: the development of a spatial analogy to teach geologic time. No details were given regarding the specific hypotheses of the intervention. The intervention and control activities were administered during a laboratory period on stratigraphy. The students in the intervention condition participated in the hierarchical alignment activity (1.5 hours) after a shortened stratigraphy laboratory (30 minutes). The guest lecturer and TA did not compare the hierarchical alignment activity and stratigraphy laboratory; they were presented as entirely separate activities. The students in the control condition completed the full stratigraphy laboratory (2 hours). Students were randomly assigned to either the intervention or control condition so that both conditions were evenly distributed across the TAs to control for instructor-based differences. All students completed the outcome measures 1 month after the stratigraphy laboratory as part of a laboratory examination.

**Fig. 2:**
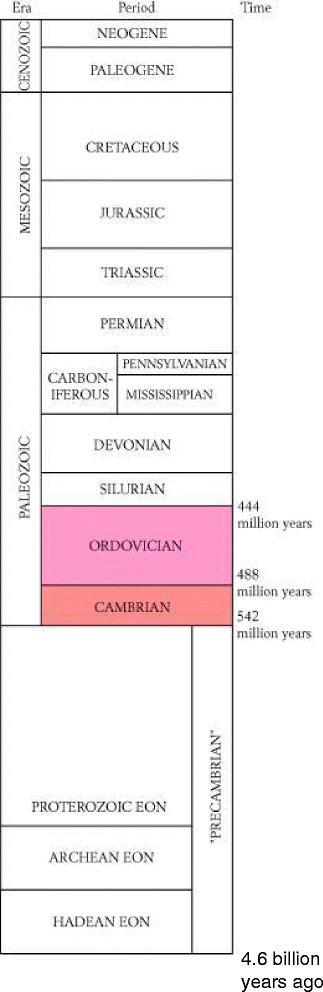
Example of conventional image of the Geologic Time Scale. Note: This image was shown to all students across Experiments 1 and 2 as part of normal course instruction. At the beginning of each lecture, the corresponding time period was highlighted

The intervention was conducted by the first author as a guest lecturer. Fidelity of implementation was achieved by having the first author develop and administer the intervention. The control activity was the full stratigraphy laboratory administered by the course TAs, as part of their normal course instruction. Both the intervention and control conditions also received instruction on the Geologic Time Scale as part of the normal class curriculum in addition to the hierarchical alignment activity and stratigraphy laboratory. Students were required to memorize the major divisions in the Geologic Time Scale. Furthermore, before beginning a lecture on a new time division, the students were shown a conventional image of the Geologic Time Scale, with the respective time division(s) highlighted (see Fig. 2). Students also learned other concepts that are explicitly related to geologic time; students completed two fossil laboratories, which included identifying fossils from different divisions in time. Thus, students across conditions had multiple opportunities to compare different representations of geologic time; the only difference being that the intervention condition had the hierarchical alignment activity whereas the control condition had additional stratigraphic problems within the stratigraphy laboratory.

## Results

**Fig. 3:**
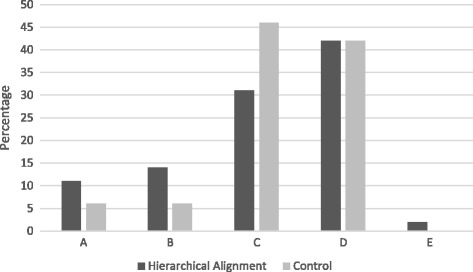
Distribution of student responses in Experiment 1 on the Geologic Concept Inventory question. Note: Students are presented with five time lines: response option A – life occurred when Earth formed, option B – humans and dinosaurs coexisted, option C – dinosaurs appeared much earlier than they did, option D – the correct relative positions, and option E – all life formed at the beginning of Earth’s history

The intervention conditions were administered in half of each TA’s sections of the course and the control conditions in the other half. No differences among the TA sections were found on any of the outcome measures and, thus, subsequent analyses compared the intervention and control conditions across TA sections. The intervention group performed better than the control group on the two items that assessed understanding of geologic magnitude. First, on the Geoscience Concept Inventory question, students in the intervention group were significantly less likely to make large magnitude errors than students in the control group, χ^2^(1) = 6.08, *p* = .01, Φ = .24. That is, they were significantly less likely to choose C, a large magnitude error and the most common error, χ^2^(1) = 7.35, *p* = .01, Φ = .26. An effect size for two binary variables (Φ) of .20 to .40 is considered a moderate association (Rea & Parker, 1992). See Fig. 3 for distribution of student responses. However, the groups did not differ in choosing the completely correct option, χ^2^(1) = .07, *p* = .79.

Second, on the Geologic Time Scale diagram question, which required students to identify the true duration-based statement using a conventional Geologic Time Scale diagram, the intervention group (37% correct) was more accurate than the control group (30% correct), χ^2^(1) = 3.99, *p* = .05, Φ = .19. In addition, students in the intervention group were less likely to select a visually misleading item that constitutes the most common error (48% of the time), whereas students from the control group selected it 69% of the time, χ^2^(1) = 4.41, *p* = .04, Φ = .20. Of importance, the intervention and control groups did not differ significantly on the knowledge-based test item, which did not require an understanding of magnitude, χ^2^(1) = .03, *p* = .86.

Transfer was assessed by the line estimation items. Here, there were no significant differences in performance between the experimental and control groups (*t*(105) = .02, *p* = .93). Failure to find differences may have resulted from the task being too easy. Mean errors (mainly overestimations) were low for both groups, ranging from .62 to 1.61 cm with standard deviations from .97 to 2.13. The observed high accuracy may have been due to the selection of items. Values near boundaries tend to show more accurate estimations than values that are distant from a boundary (Haun, Allen, & Wedell, 2005; Huttenlocher, Hedges, & Prohaska, 1988; Shipley & Zacks, 2008); six million and 65 million are both close to the beginning of the geologic number line, and 3.5 billion is close to 3/4 of the number line (breaking the number line up into quadrants). Thus, while these items were designed to be aligned with the geologic events in the Geoscience Concept Inventory item, in their numerical form they may not have been ideal for capturing variance in the underlying representations.

## Discussion

Students who completed the hierarchical alignment activity demonstrated a better sense of the relative durations of geological events and a reduction in the magnitude of temporal location errors relative to the control group on the two items assessing geologic time. Of importance, the intervention and control groups did not differ significantly on the knowledge-based item, indicating that the hierarchical alignment activity acted on the understanding of the magnitude of geologic time, and did not necessarily simply increase effort or motivation. Notably, these effects were evident *1 month* after the intervention, clearly indicating the effect was durable. Nevertheless, these positive findings need to be tempered with the facts that the intervention, although more effective than regular class instruction, did not take students to desirable levels of accuracy.

Given the context of a geology classroom we were unable to systematically align two aspects of the intervention condition (hierarchical alignment) and control condition (stratigraphy laboratory only). A guest lecturer presented the intervention condition; however, due to scheduling constraints, the regular TA presented the control condition. This may have resulted in other unaccounted differences between the intervention and control conditions. For example, the guest lecturer may have introduced a novelty as well as been more motivated to have the students learn from their carefully designed intervention. The hierarchical alignment activity also contained information on a student’s personal time line, a human lifespan, American history, and human evolution that was not covered in the control condition. Arguably, this may have benefited the control condition, because they spent more time working with the geologic time periods they were ultimately assessed on. However, this was not the case; beginning with non-course content improved students’ understanding of geologic time divisions despite spending less time working with them. Regardless, our findings were consistent with Resnick, Newcombe, *et al*. (2016), who were able to more tightly control for such differences in their laboratory-based assessment of the hierarchical alignment activity, and found the hierarchical alignment activity was effective for fostering more accurate reasoning about phenomena at extreme scales.

Another limitation of Experiment 1 was the limited measures for assessing understanding of geologic time (two items) and the high accuracy on the items selected for the line estimation task. Thus, one option for further investigation would be to replicate the intervention in Experiment 1 with more sensitive dependent variables. However, there was an additional, more practical, concern: a 1.5 hour intervention would be impractical for wide adoption in an already packed curriculum. Therefore, in Experiment 2, we had three goals:

1. Develop and test a spatial analogy that includes multiple opportunities to align time to space while maintaining structural alignment (as in Experiment 1).
2. Develop and test an activity that could be more easily integrated into classrooms.
3. Develop more sensitive measures.

## Experiment 2

Experiment 2 was conducted over 2 years in the same introductory-level geoscience course as in Experiment 1. In Experiment 2, the course was taught by two instructors as separate classes. The instructors worked together to make the two classes as similar as possible; they developed materials together (slides, lectures, curricular sequence, exercises, and examinations), followed the same schedule of topics, and used the same textbooks and laboratory activities. However, there was a critical difference in the way in which they administered the spatial analogy activity developed for assessment here (detailed below). Subsequently, we can conceptualize the interventions administered in either class as separate interventions, which we will refer to as the *clicker feedback activity* and the *linear visualization activity*. All comparisons between the intervention and control are thus made *within* activity/instructor and *not* between activities/instructors. Taken together, the results from Experiments 1 and 2 provide evidence of the effect of spatial analogies using a structural, progressive, and hierarchical alignment paired with corrective feedback, as instantiated within each instructor’s teaching style.

The clicker feedback activity and the linear visualization activity both share many features with the hierarchical alignment activity assessed in Experiment 1. All the activities provide multiple opportunities to align time to a spatial linear representation using the same amount of space for each alignment (structural alignment) and provide students with corrective feedback. Of importance, the clicker feedback activity and linear visualization activity differ from the hierarchical alignment activity by not progressing from small familiar scales to geological scales (progressive alignment) and not hierarchically organizing all previous scales within the current scale (hierarchical alignment). Rather, the clicker feedback activity and the linear visualization activity ask students to align the divisions of the Geologic Time Scale to a linear scale in their sequential order. In addition, the exercises offered spaced practice in time estimation, which is important because spacing effects are a well-established principle in the optimization of learning (Pashler *et al*., 2007).

In the clicker feedback activity students align the non-linear representation of the Geologic Time Scale from their textbook with a linear time line, and are provided with corrective feedback using the clicker response system (Turning Technologies, LLC, 2013). The clicker response system is a handheld electronic device that can be used to answer multiple choice questions. Such electronic devices have been found to improve learning and engagement, particularly when paired with immediate feedback (Kay & LeSage, 2009). Submitting a clicker response involves the student in making a specific prediction, which is then confirmed or disconfirmed; this is a process that has also been found to improve understanding (for example, Howe, Rodgers, & Tolmie, 1990).

In the linear visualization activity, only the corrective feedback information about the Geologic Time Scale was presented visually as a linear representation. That is, students did not make a prediction about how the Geologic Time Scale from their textbook would align with the linear time line. Rather, they saw only the final image that aligned the two scales. They did not use the clicker response system.

More, and more sensitive, measures were also developed for Experiment 2. Twelve items were designed to assess reasoning about geologic content that required magnitude knowledge. A more sensitive number line task was developed, using a spatially longer time line and numbers farther away from salient boundaries. Finally, examination-level performance and demographic information were obtained for the sample.

## Methods

### Participants

The experiment took place during the Fall 2011, Spring 2012, and Fall 2012 semesters at a large urban American university. Participants consisted of students enrolled in an undergraduate introductory-level geoscience course aimed primarily at non-majors, who attended at least seven of the ten classes covering the Geologic Time Scale (the last ten lectures). These inclusion criteria were used to avoid including students who had little experience with the intervention. A total of 625 students were registered for the two courses over three semesters. There were 229 students who attended at least 70% of classes out of 300 registered in the clicker feedback study: 75 (Fall 2011) in the control condition, 74 (Spring 2012) and 80 (Fall 2012) in the intervention conditions. There were 198 students who attended at least 70% of classes out of 325 registered in the linear visualization study: 61 (Fall 2011) in control condition, 50 (Spring 2012) and 87 (Fall 2012) in the intervention conditions.

A majority of the students (71%) who did not meet the inclusion criteria (attendance of seven of the ten classes) were absent for more than half of the classes. This offers some insight into the amount of exposure to the intervention for the students who were not included in the analysis: the students did not meet the inclusion criteria by a large margin because they were absent for the majority of the intervention content. This attendance rate was consistent across the control and intervention conditions.

### Procedure

In Fall 2011, students received regular instruction (control condition) in both study types (clicker feedback and linear visualization). Regular instruction was administered primarily through lectures employing PowerPoint slide presentations. Geologic events were taught over approximately ten class sessions. Lectures on geologic events were organized by temporal divisions in the Geologic Time Scale (for example, the first two lectures were on the Precambrian Eons, with the subsequent lectures covering portions of Eras within the Phanerozoic Eon). As in Experiment 1, at the beginning of each new lecture, the image of the Geologic Time Scale from the class’s textbook was presented as an introduction, with the relevant division in time highlighted (see Fig. 2). Of importance, the image presents the time scale in a vertically condensed format. That is, in the image time to space is not mapped linearly; the Precambrian (roughly 4 billion years) is compressed to the bottom of the time line, and the Phanerozoic Eon (542 million years), which is not labeled in Fig. 2, is expanded to show the individual Periods of each Era (the Paleozoic, Mesozoic, and Cenozoic). This representation of the Geologic Time Scale is conventional and functional as it allows users to see the smaller divisions of time in the Phanerozoic. Both teachers estimated that this introductory slide was presented for 10 seconds at the beginning of each of the ten lectures.

After learning about geologic events in the ten class sessions, students were tested on this material on the third examination in the course, which did not contain material from the first two sections, in the 11th class session. The three multiple choice items used in Experiment 1 were added to the regular examination, as well as 12 multiple choice items and four line estimation items to assess magnitude representation and reasoning (see Measures section below).

**Fig. 4:**
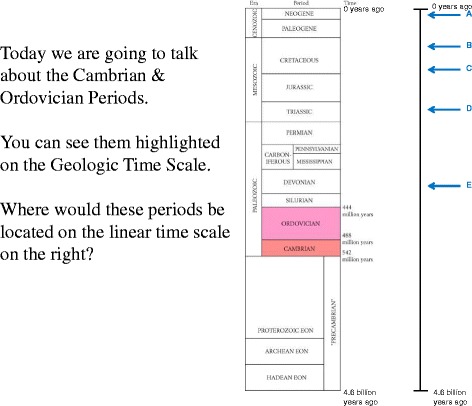
Example of initial slide from the clicker feedback activity. Note: Students predict where the highlighted section on the conventional image of the Geologic Time Scale would be located on a linear scale

**Fig. 5:**
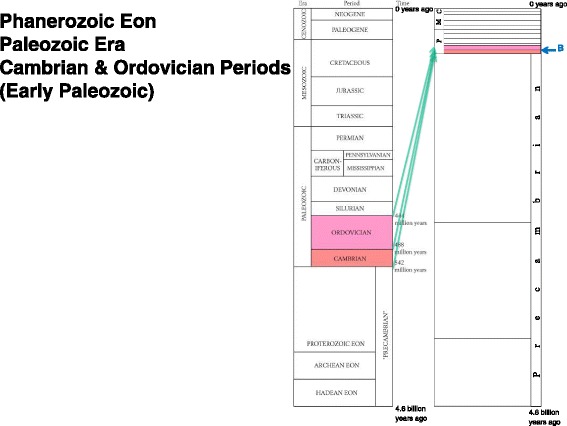
Example of slide aligning the conventional Geologic Time Scale to a linear scale

In the clicker feedback activity, the introductory slide with the Geologic Time Scale image was replaced with two PowerPoint slides. The first slide was identical to the Geologic Time Scale image used in the baseline instruction; however, there was also a blank time line to the right of the original image (see Fig. 4). Four locations on the blank time line were labeled A through D. Students were asked which of the four response options showed where the middle of the highlighted division in time would be located on the linear scale. Students responded by using the clicker response system. Once students responded, the instructor presented the second slide with the answer, thus giving the students corrective feedback (see Fig. 5). On the corrective feedback slide, the original image was presented, along with an image of a linear representation of the Geologic Time Scale. The relevant divisions in time were highlighted on both images and arrows connected the highlighted divisions in time on the two time lines. The instructor estimated the presentation of both slides took on average 30 seconds to present at the beginning of each of the ten lectures, with the first two presentations requiring more time because students were given initial instructions and explanation of what the image represented.

In the linear visualization activity, students were also shown two slides. The first slide was the original slide from the control condition (Fig. 2). The second slide is the same as the feedback slide in the clicker condition, where the conventional image of the Geologic Time Scale is aligned with a linear representation (see Fig. 5). Thus, students were not asked to predict how the conventional image aligned with a linear representation and then given corrective feedback; rather, they were simply shown the correct relationship between the two time lines. The instructor estimated the presentation of the slide took on average 10 seconds at the beginning of each of the ten lectures, with the first two presentations requiring more time because students were given initial explanations of what the images represented.

A number of procedures were used to achieve fidelity of implementation. The control condition was regular course instruction, and occurred in the course (Fall 2011) prior to when the intervention was administered (Spring 2012 and Fall 2012). After the control semester was completed, and prior to beginning the intervention, the first author met with the teachers to go through the intervention materials. Next, the first author obtained the lecture slides from the teachers from the Fall 2011 course, inserted the intervention slides, and sent them back to the teachers. The teachers were contacted again immediately prior to the first intervention session to remind the teachers of the protocol. Finally, after the course was finished, the first author met with the teachers to debrief on their implementation and experiences with the intervention. The teachers’ self-report was consistent with the procedure outlined above.

### Measures

The three multiple choice items used to assess magnitude representation in Experiment 1 were also included here. In addition, a set of 12 multiple choice items were developed to assess understanding of scientific concepts involving large temporal scales. Eight items required only recall of temporal magnitudes (for example, “When did dinosaurs appear?”). To measure differences in recall of categorical and metric information, four items included categorical options (for example, “A. Triassic…), and four included numeric magnitude response options (for example, “A. 230 million years ago…). Note that 230 million years ago was within the Triassic. Two counter-balanced examination versions were created. Each version had two categorical response items and two metric response items. Thus, students received only four ‘recall only’ questions. This between-subjects design was adopted because of limited space on the examination and to avoid the unusual repetition of items in a classroom examination that would be required for a within-subject design.

Four additional items were developed that required magnitude recall plus an additional step of comparison (for example, “What is the relationship between dinosaurs disappearing and humans appearing?”). In this example, participants are required to recall when dinosaurs disappeared, when humans appeared, and compare the relative duration in between the two events. All students received all four of these items.

To measure representation of abstract (numeric) magnitude, independent of any content-specific information, a series of line estimation tasks were given. Participants were given a sentence stating when an event occurred, and then asked to locate that magnitude on the number line (for example, “The Rocks of Sierra Nevada Mountains formed between 125 million years ago and 85 million years ago. Draw on the time line provided when these rocks formed.”). These items were framed in terms of events to match the form of the other experimental measures. These estimations are considered estimations of abstract magnitude because the participants are explicitly given a magnitude to place on the number line; no recall is required. To assess representations of the million and billion scales, participants were asked to estimate two “events” on a 4.6 billion scale, and two on a 542 million scale. Because, in Experiment 1, the relatively low error on the abstract magnitude estimation may have been due to magnitudes being located near salient boundaries (for example, 65 million on a 4.6 billion scale is very close to the beginning of the number line), in Experiment 2, we specifically used magnitudes located farther away from the ends of the number line. The line estimation task was scaled to a 173.5 mm vertical line.

Three non-cumulative examinations were given during the semester; examination grades on each were included in our analyses. Examinations one and two were given prior to the intervention activities. Examination three grades reflect the relative effectiveness of the intervention activities (clicker feedback and linear visualization). Absences for the semester were collected. Demographic information was obtained through the General Education Office of the University.

## Results

### Examination performance prior to intervention

**Fig. 6:**
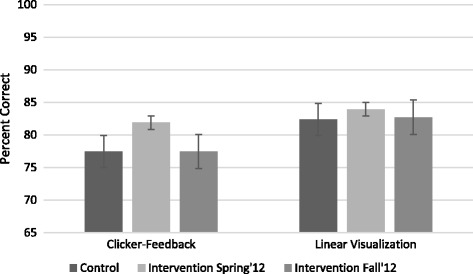
Mean examination performance (exams 1 and 2) prior to content on the Geologic Time Scale

To examine if there were differences on performance on examinations given prior to intervention, and covering material not concerned with geologic time, an ANOVA was conducted for the average score of examinations one and two (measure of pre-intervention performance) with factors of semester (Fall 2011 control, Spring 2012 intervention, Fall 2012 intervention) and study type (clicker feedback versus linear visualization). See Fig. 6 for means of the pre-intervention examinations by study type and semester. Main effects of semester (F(2,420) = 5.56, *p* = .004, ${\eta}_p^2=.03$) and study type (F(1,420) = 24.93, *p* < .001, ${\eta}_p^2=.06$) were found, but no interaction (F(2,420) = 2.19, *p* = .113). The measure of effect size here $\left({\eta}_p^2\right)$ is interpreted as the proportion of the ratio of variance accounted for by an effect and that effect plus its associated error variance within an ANOVA (Cohen, 1973). Thus, semester accounts for 3% and study type accounts for 6% of the variance in examination performance, which are relatively small effect sizes.

Individual comparisons between semesters and between study type were assessed using Bonferroni adjusted alpha levels of .0125 per test (.05/4 comparisons). Students did better in the Spring 2012 semester compared with the Fall 2011 control group (*t*(423) = 2.55, *p* = .011, *d* = .33) and the Fall 2012 semester intervention (*t*(423) = 2.81, *p* = .005, *d* = .33). An effect size comparing the difference in means between two groups (*d*) of .2 is considered small, .5 medium, and .8 a large association (Cohen, 1988). The two Fall groups performed similarly on the pre-intervention measures (*t*(423) = .14, *p* = .89). Both instructors have noted this pattern of slightly lower performance in the Fall semester compared with the Spring semester over the course of their experience teaching these classes.

Students who would receive the clicker feedback activity had a significantly lower score on pre-intervention examinations than those who were slated to receive the linear visualization activity (*t*(424) = 4.95, *p* < .001, *d* = .48). As there was no interaction, this difference also applied to the Fall 2011 control group, and may result from differences in the kind of students enrolled in the classes taught by the two instructors based on other scheduling considerations. We controlled for this difference in analyses of the intervention, using ANCOVA.

### Was the intervention successful in improving examination scores?

To examine if there were differences on examination performance after the intervention, a 3 (semester – Fall 2011 control, Spring 2012 intervention, Fall 2012 intervention) by 2 (clicker feedback and linear visualization) ANCOVA was conducted. Each student’s average performance on their two pre-intervention examinations was included as a covariate. Main effects of semester (*F*(2,420) = 37.17, *p* < .001, ${\eta}_p^2=.15$) and study type (*F*(1,420) = 95.32, *p* < .001, ${\eta}_p^2=.19$ were found. Of importance, there was also a significant interaction between condition and study type (*F*(2) = 11.58, *p* < .001, ${\eta}_p^2=.05$).

**Fig. 7:**
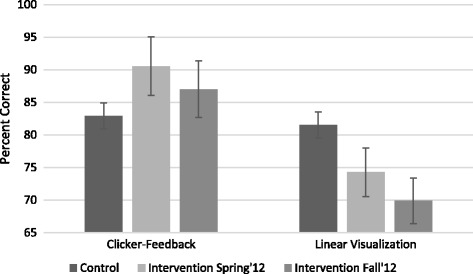
Mean examination performance (exam 3) on content covering events on the Geologic Time Scale

Individual comparisons between semesters and between study type were assessed using Bonferroni adjusted alpha levels of .0125 per test (.05/4 comparisons). Students who received the clicker feedback activity had significantly higher scores on examination three than the corresponding Fall 2011 control condition: Spring 2012, *t*(225) = 3.6, *p* < .001, *d* = .58; Fall 2012, *t*(225) = 4.53, *p* < .001, *d* = .31 (see Fig. 7 for means). On the 50-question examination, 82% of the examination questions in the clicker feedback study were time-relevant questions; that is, they related to the ordering of geologic events in time or related to a specific period in geologic history. Thus, the improved examination performance suggests the clicker feedback activity improved understanding of scientific concepts associated with large temporal scales.

On the other hand, students who received the linear visualization activity performed significantly worse on examination 3 than the corresponding Fall 2011 control condition (Spring 2012, *t*(195) = 2.96, *p* = .004, *d* = .57; Fall 2012, *t*(195) = 5.42, *p* < .001, *d* = .87), suggesting that exposure to the linear visualization activity actually interferes with learning. In the linear visualization study, 80% of the examination questions were time-relevant questions. This small variation in questions represents a slightly different focus of either instructor on additional material. However, analysis conducted on just overlapping questions results in the same findings as the presented analysis on the examination as a whole.

### Was the intervention successful in improving reasoning about geologic content that requires an understanding of magnitude?

On the Geologic Concept Inventory item (which measures temporal magnitude representation), students who received the clicker feedback activity were more likely to choose the correct response option than the Fall 2011 control condition (% correct = 62): Spring 2012, % correct = 76, χ^2^(1) = 3.64, *p* = .04, Φ = .16; Fall 2012, % correct = 73, χ^2^(1) = 3.5, *p* = .05, Φ = .15. An effect size for two binary variables (Φ) of .10 to .20 is considered a weak association (Rea & Parker, 1992). Students who received the linear visualization activity were not significantly different from the control condition on this item (% correct = 63): Spring 2012, % correct = 60, χ^2^(1) = .15, *p* = .70; Fall 2012, % correct = 65, χ^2^(1) = .04, *p* = .84.

Students completed 12 additional multiple choice items designed for this study to measure knowledge and reasoning about temporal magnitude. For eight of the items, students were asked to identify a time-based fact that was taught in class (for example, “When did dinosaurs appear”). For the four remaining items, students were asked to judge relative duration of an event (for example, “How far do continental plates move in a single year”). The content of these items had not been explicitly taught in class; they required inferences from information that was taught. There were no significant differences detected between study type (clicker feedback and linear visualization) or between semesters (Fall 2011 control, Spring 2012 intervention, Fall 2012 intervention) on any of these multiple choice items (*p* > .05).

### Was the intervention successful in producing transfer to abstract magnitude?

**Fig. 8:**
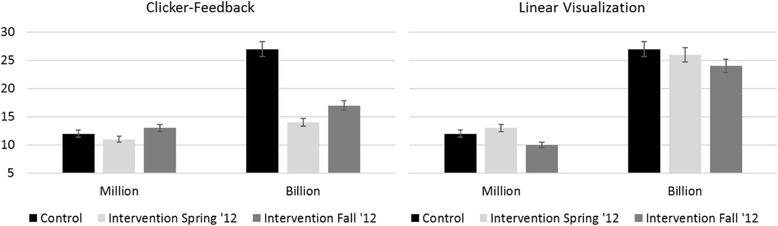
Mean error in abstract magnitude number line estimations (error bars = standard deviation)

Students across study type and semester were more accurate on estimations at the million scale (μ error = 11.73 mm) than the billion scale (μ error = 22.87 mm): (*t*(343) = 10.19, *p* < .001, *d* = .58). The million scale is arguably now within the range of common human experience (for example, housing prices), and thus more familiar to students than the billion scale. Given this relative accuracy on the million scale, it is unsurprising that there were no significant differences between study type (*t*(344) = .13, *p* = .90) or between semesters (*F*(1,344) = .12, *p* = .74) at estimating magnitudes at this scale. However, at the billion scale, students who received the clicker feedback activity were significantly more accurate than those in the Fall 2011 control condition at estimating abstract magnitudes (Spring 2012, *t*(179) = 3.5, *p* = .05, *d* = .58; Fall 2012, *t*(179) = 2.02, *p* = .04, *d* = .39), and more accurate than those students who received the linear visualization activity (t(246) = 2.22, *p* = .027, *d* = .3). Notably, the average error on the billion scale following the clicker intervention was comparable to the error seen on the millions scale. There was no significant difference between students who received the linear visualization activity and the control condition on this measure (Spring 2012, *t*(165) = .25, *p* = .80; Fall 2012, *t*(165) = .93, *p* = .35). See Fig. 8 for means of abstract magnitude line estimations.

## Discussion

The clicker feedback activity in Experiment 2 was associated with improved examination scores, and more linear representations of temporal and abstract numeric magnitudes, relative to students who received normal class instruction. It is important to note here that while an exam-level effect may be surprising, the course content for the final examination was structured around events on the Geologic Time Scale; subsequently, understanding the relationship between geologic events on a linear scale is directly relevant for examination performance. In addition, we find this relationship between the corrective feedback activity and examination performance across two semesters, showing this is a replicable effect. Understanding the scale of geologic time may provide a context in which to structure key course concepts, such as major evolution radiations, plate tectonics, and climate changes; subsequently, students are better able to understand underlying geologic processes and systems.

On the other hand, the linear visualization activity was associated with *lower* examination scores than normal class instruction, and no improvement on abstract magnitude. This outcome can be understood from the perspective of research on multiple external representations, which suggests that failure to align different representations can interfere with understanding (Ainsworth, 1999). Aligning different representations can be difficult for learners (de Jong *et al*., 1998), and making such alignments is characteristic of expert understanding (Kozma, Chin, Russell, & Marx, 2000). The linear visualization activity presented only the conventional image of the Geologic Time Scale aligned with a linear representation (see Fig. 5). It is possible that students in the linear visualization study failed to make the correct alignment. Consequently, the linear visualization students may not have understood the relationship between the two time lines; having multiple representations of what appeared to be the same information may have interfered with overall understanding. This interpretation is consistent with the finding that students who received the linear visualization activity were not significantly different from baseline (pre-intervention) on estimations of abstract magnitudes at the billion scale: there was no evidence of alignments between the compressed image of the Geologic Time Scale and the linear time line. By contrast, the clicker feedback activity actively engaged the students in making the alignment by requiring them to predict the relation using the clicker response system.

There were no significant differences in performance on the multiple choice items added to the examination. However, transfer was seen to large abstract magnitude reasoning as indexed by the number line task. Perhaps the clicker feedback activity helps primarily by placing course material into context and scale, thus, developing more linear representations of magnitude; however, novices were reluctant to estimate when unknown events occurred on the Geologic Time Scale. They may approach questions asking when an event occurred as requiring a precise answer that they either know, in which case they chose the correct answer, or they do not know, in which case they randomly pick an answer. Although the nature of geoscience is to use current day spatial configurations of strata (rock layers) to estimate a sequence of events (Parcell & Parcell, 2009), and experts use knowledge to make estimations of unknown magnitudes, it is possible that instruction has not yet led novices to try to estimate time scales from other knowledge. In addition, the format of the question (that is, discrete response options) may have promoted a “know it-or-guess” approach. An open-ended format, such as number line estimations, might help promote estimation-based responses by novices, allowing future research to examine magnitude representation in estimation of scientific phenomenon.

## Conclusions

The findings from our two studies suggest teaching scale information using structural, progressive, and hierarchical alignment paired with corrective feedback, is effective for the development of a linear representation of magnitude. In particular, providing students with multiple opportunities to align magnitude relations on a spatial linear scale appears to be a critical component of learning. Of importance, as seen with the linear visualization activity, simply seeing the Geologic Time Scale aligned to a spatial linear representation may not be enough to develop a linear representation of geologic magnitude. Students with a more accurate understanding of the Geologic Time Scale relative to a control had actively engaged in constructing a linear representation. This finding is consistent with previous work showing the process of prediction and feedback improves understanding (Howe *et al*., 1990). While hierarchical alignment and progressive alignment are effective techniques, the success of the clicker feedback activity suggests they may not be necessary in learning about scale. However, Resnick and Shipley (2013) found that there might be an additive benefit for hierarchical and progressive alignment over progressive alignment alone. Future research is required to examine the relative contributions of these analogical principles (structural, progressive, and hierarchical alignment): are there certain contexts in which certain principles are more effective than others, and are there additive effects for increased learning? In addition, how do these analogical principles interact with active prediction, spaced feedback, and other kinds of visual representations in learning scale information?

A main limitation of Experiment 1 was the small number of items that assessed understanding and reasoning about geologic time. While Experiment 2 includes a larger range of assessment items, future research is required to develop more sensitive items to examine how people reason about phenomena outside human perception. For example, are there systematic differences on discrete (for example, multiple choice) versus continuous (for example, number line estimation) questions on reasoning about magnitude?

That learning about temporal magnitudes in the clicker feedback activity transferred to abstract magnitude representation suggests magnitudes across domains share at least some common features (consistent with Walsh, 2003). This finding is particularly important for educators across STEM disciplines, as understanding size and scale is essential in understanding a range of scientific concepts and has been identified as a fundamental and unifying theme in science education by the National Research Council in *A Framework for K-12 Science Education* (National Research Council, 2011) and the American Association for the Advancement of Science in *Benchmarks for Science Literacy* (American Association for the Advancement of Science, 1993). A future program of research should examine if a spatial analogy activity could serve as a way to build a foundation of scale understanding. For example, using the clicker feedback activity for a range of scales and contents may help students make connections between a wide range of phenomena and magnitudes, and align the vast set of scales across the sciences.
